# Supplementary material for: Mycobacterium tuberculosis SatS is a chaperone for the SecA2 protein export pathway
Source: eLife. 2019 Jan 3;8:e40063. doi: 10.7554/eLife.40063 (PMC6333443; doi:10.7554/eLife.40063)
Supplement: Figure 8—source data 1. [file elife-40063-fig8-data1.docx]

SatS_C_ X-ray Structure Validation Details.

|  | **SatS_C_(Br)** | **SatS_C_(Apo)** |
| --- | --- | --- |
| **PDB ID** | 6DRQ | 6DNM |
| **Data collection** |  |  |
| Space group | P 2­_1_2_1_2_1_ | P 2­_1_2_1_2_1_ |
| Cell dimensions |  |  |
| *a, b, c* (Å) | 48.8, 50.6, 76.1 | 48.9, 50.6, 76.5 |
| *α, β, γ* (°) | 90, 90, 90 | 90, 90, 90 |
| Resolution (Å) | 2.3 | 1.4 |
| *R_merge_* | 0.050 (0.105) | 0.081 (0.254) |
| *I/σ(I)* | 27.4 | 14.4 |
| Completeness (%) | 99.2 (100) | 92.6 (70.4) |
| Redundancy | 7.0 (7.3) | 5.9 (1.4) |
|  |  |  |
| **Refinement** |  |  |
| Resolution (Å) | 42.129-2.3 | 21.19-1.4 |
| No. reflections | 8721 | 35457 |
| *R_work_/R_free_* | 0.2095/0.2973 | 0.193/0.231 |
| No. atoms |  |  |
| Protein | 1463 | 1467 |
| Ligand/ion | 3 |  |
| Water | 13 | 110 |
| B-factors |  |  |
| Protein | 40 | 19.9 |
| Ligand/ion | 44 |  |
|  |  |  |
| R.m.s. deviations |  |  |
| Bond lengths (Å) | 0.007 | 0.005 |
| Bond angles (°) | 0.867 | 0.736 |
